# Supplementary material for: Inferring age-specific differences in susceptibility to and infectiousness upon SARS-CoV-2 infection based on Belgian social contact data
Source: PLoS Comput Biol. 2022 Mar 30;18(3):e1009965. doi: 10.1371/journal.pcbi.1009965 (PMC9000131; doi:10.1371/journal.pcbi.1009965)
Supplement: S1 Appendix — Presentation of the complete results with all figures and tables containing values. Table A: Timetable of CoMix starting dates. Fig A. Schematic timeline of CoMix waves. Table B: Timetable of Belgian testing policy. Figs. B and C. Sensitivity analysis of the method under different assumptions. Figs. D to S: Estimates of relative proportionality factors and corresponding relative incidence under different assumptions. Table C to J: Exact estimates of the components of the proportionality factors. (PDF) [file pcbi.1009965.s001.pdf]

# S1 Appendix: Supplementary Material

In this Supplementary Material, we present additional results on the estimation of heterogeneous proportionality factors using the next generation matrix as well as some timeline descriptions and a sensitivity analysis to assumptions.

Social contact data $\mathbf{M}$ are taken from CoMix waves 12 to 23 as described in the timetable given in Table A and timeline in Fig A and PCR tests were performed under the testing policy presented in Table B. Relative incidences are computed as the leading eigenvector of the next generation matrix $\text{diag}(a_i)\,\mathbf{M}^T\,\text{diag}(h_j)$ and compared with the normalized relative incidence estimated from PCR positive tests. The $q$-susceptibility $(a_i)$ and $q$-infectiousness $(h_j)$ vectors are either assumed or estimated. A sensitivity analysis concerning the assumptions on $(a_i)$ and $(h_j)$ vectors is presented in Figs B and C.

In the last subsections, we present the complete details of 4 different sets of results:

- Estimation of $q$-susceptibility $(a_i)$ using homogeneous infectiousness assumption $h_j = 1\ \forall j$ (Figs D to G and Tables C and D)
- Estimation of $q$-susceptibility $(a_i)$ using heterogeneous infectiousness assumption $(h_j)$ coming from the literature (Figs H to K and Tables E and F)
- Estimation of $q$-infectiousness $(h_j)$ using homogeneous susceptibility assumption $a_i = 1\ \forall i$ (Figs L to O and Tables G and H)
- Estimation of $q$-infectiousness $(h_j)$ using heterogeneous susceptibility assumption $(a_i)$ coming from the literature (Figs P to S and Tables I and J)

Each time, we present the estimate of the proportionality factors for the complete period (waves 12 to 23) or estimated by groups of two waves, with the exact values presented in the subsequent tables. The normalization method is described at the beginning of each subsection. We associate to the results the estimate of the relative incidence, with the real data presented in blue, the estimate without any proportionality factor $a_i = h_i = 1\ \forall i$ in green and the estimate with the assumed and estimated proportionality factors in red. Dots represent means and bars represent 95% nonparametric bootstrap confidence intervals (2.5% and 97.5% quantiles).

## CoMix and PCR test timelines

We present here details concerning Belgian timelines on social contact data collection and PCR testing policy. A detailed timetable of the CoMix waves and survey periods is presented in Table A. The period of study is restricted on CoMix waves 12 to 23 since it corresponds to an identical survey design. A schematic timeline of CoMix waves according to the evolution of the alpha variant of concern and vaccination campaign in Belgium is presented in Fig A. This period of study also corresponds to an almost constant testing policy, with mandatory testing both symptomatic cases and asymptomatic close contacts and travelers and before biases induced by the introduction of the EU Digital COVID Certificate. A summary of Belgian testing policy is presented in Table B though more information can be found in [1].

| Survey 1 | | Survey 2a | | Survey 2b | |
|---|---|---|---|---|---|
| wave 1 | 20 April 2020 | wave 9 | 11 November 2020 | wave 12 | 22 December 2020 |
| wave 2 | 5 May 2020 | wave 10 | 25 November 2020 | wave 13 | 6 January 2021 |
| wave 3 | 18 May 2020 | wave 11 | 9 December 2020 | wave 14 | 20 January 2021 |
| wave 4 | 1 June 2020 | | | wave 15 | 3 February 2021 |
| wave 5 | 15 June 2020 | | | wave 16 | 17 February 2021 |
| wave 6 | 29 June 2020 | | | wave 17 | 3 March 2021 |
| wave 7 | 13 July 2020 | | | wave 18 | 17 March 2021 |
| wave 8 | 27 July 2020 | | | wave 19 | 31 March 2021 |
| | | | | wave 20 | 14 April 2021 |
| | | | | wave 21 | 28 April 2021 |
| | | | | wave 22 | 12 May 2021 |
| | | | | wave 23 | 26 May 2021 |

**Table A.** Timetable of CoMix with starting dates. The first survey (waves 1-8) did not include children. The subsequent waves included children with a preliminary version of the survey (waves 9-11), which is modified and finalized in December 2020 (waves 12-...).

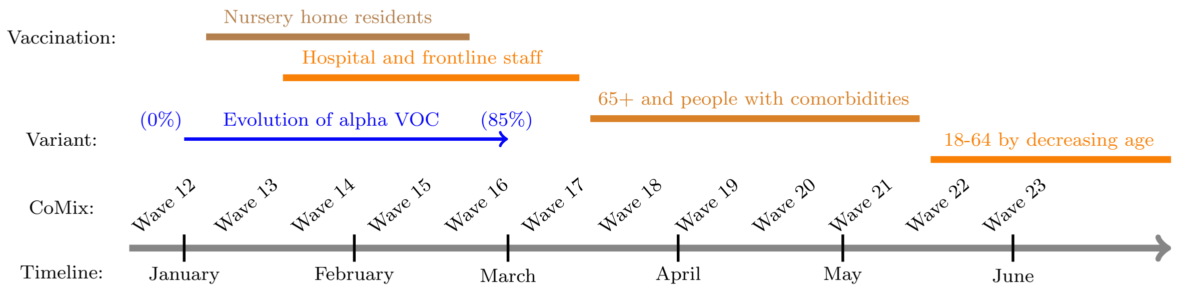


**Fig A.** Schematic timeline of CoMix waves, vaccination campaign in Belgium and evolution of alpha VOC.

| Date | Change in testing policy |
|---|---|
| 05 April 2020 | Start of mandatory testing of all symptomatic cases with PCR tests |
| 06 June 2020 | Start of mandatory testing of all asymptomatic close contacts with PCR tests |
| 21 October 2020 | Temporary stop of testing of all asymptomatic close contacts |
| 22 November 2020 | Restart of mandatory testing of all asymptomatic close contacts |
| 31 December 2020 | Mandatory testing of all red zone travelers (2 PCR tests: days 1 and 7) |
| 25 January 2021 | Asymptomatic close contacts must perform 2 PCR tests (days 1 and 7) |
| 16 June 2021 | Antigen and PCR tests are available for EU Digital COVID Certificates |

**Table B.** Summary of the main changes in Belgian testing policy.

## Sensitivity analysis

Since our method depends on assumptions made on $q$-susceptibility $(a_i)$ or $q$-infectiousness $(h_j)$ vectors, we present an additional sensitivity analysis concerning those assumptions. This analysis is performed by fixing a single bootstrap (hence removing the uncertainty related to social contact data) but varying the assumption on the $q$-infectiousness vector $(h_j)$ or the $q$-susceptibility vector $(a_j)$. We start with the heterogeneous infectiousness assumption $(h_j) = (0.54, 0.55, 0.56, 0.59, 0.7, 0.76, 0.9, 0.99, 0.99, 0.99)$ or $(a_j) = (0.4, 0.39, 0.38, 0.79, 0.86, 0.8, 0.82, 0.88, 0.74, 0.74)$ and perform a random uniform additive variation $\mathcal{U}[-0.1, 0.1]$ on each of the 10 components. Figure B represents the complete variation of the estimate on relative $q$-susceptibility $(a_i)$ (analysis similar to Fig H) under 200 simulated random variations on $(h_j)$ while Figure C represents the complete variation of the estimate on relative $q$-susceptibility $(h_i)$ (analysis similar to Fig P) under 200 simulated random variations on $(a_j)$.

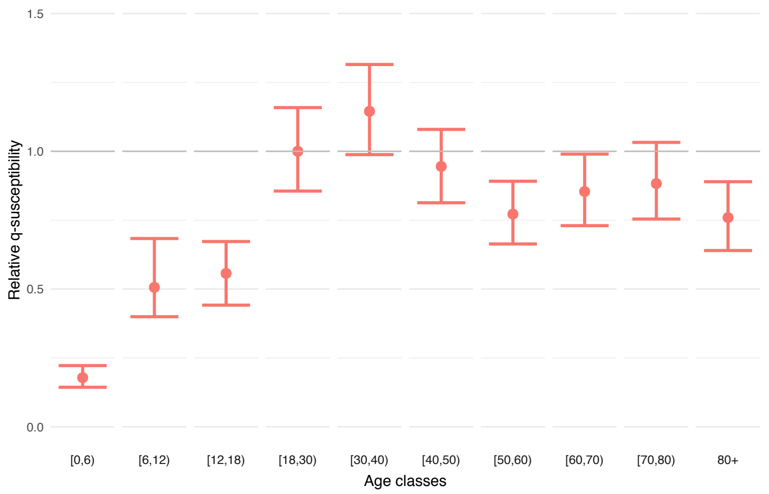


**Fig B.** Sensitivity analysis on the estimation of relative $q$-susceptibility under random heterogeneous uniform variation $\mathcal{U}^{10}_{[-0.1,0.1]}$ on the assumption on infectiousness $(0.54, 0.55, 0.56, 0.59, 0.7, 0.76, 0.9, 0.99, 0.99, 0.99)$.

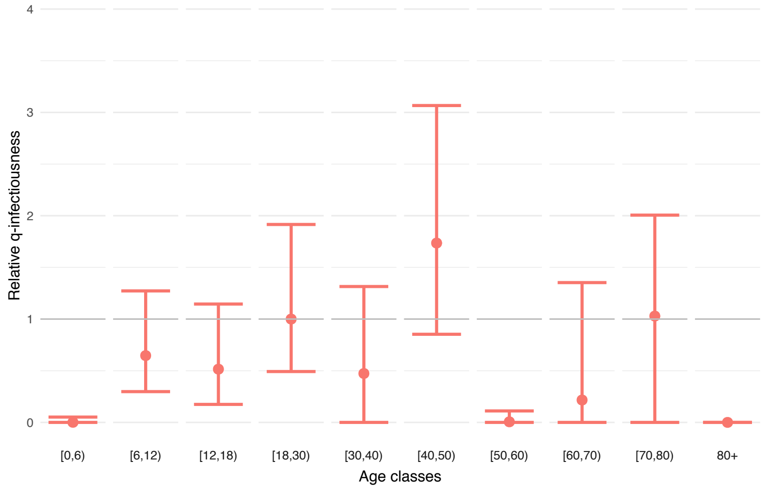


**Fig C.** Sensitivity analysis on the estimation of relative $q$-infectiousness under random heterogeneous uniform variation $\mathcal{U}^{10}_{[-0.1,0.1]}$ on the assumption on susceptibility $(0.4, 0.39, 0.38, 0.79, 0.86, 0.8, 0.82, 0.88, 0.74, 0.74)$.

## Estimation of $q$-susceptibility using homogeneous infectiousness

Method: Estimation of the $(a_i)$ relative $q$-susceptibility vector.

Assumption: homogeneous infectiousness $(h_j) = (1, 1, 1, 1, 1, 1, 1, 1, 1, 1)$.

Normalization method: Mean $q$-susceptibility among children age classes [0,6), [6,12) and [12,18) is assumed constant among bootstraps and wave groups (if applicable). The mean of the first adult age class [18,30) is set to 1 for the first period.

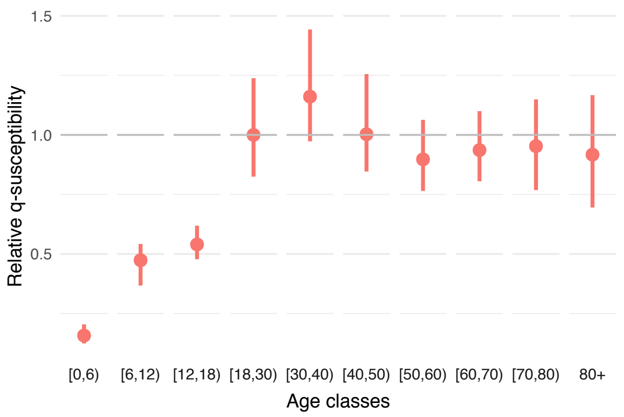


**Fig D.** Estimation of relative $q$-susceptibility using assumption on infectiousness $(1, 1, 1, 1, 1, 1, 1, 1, 1, 1)$.

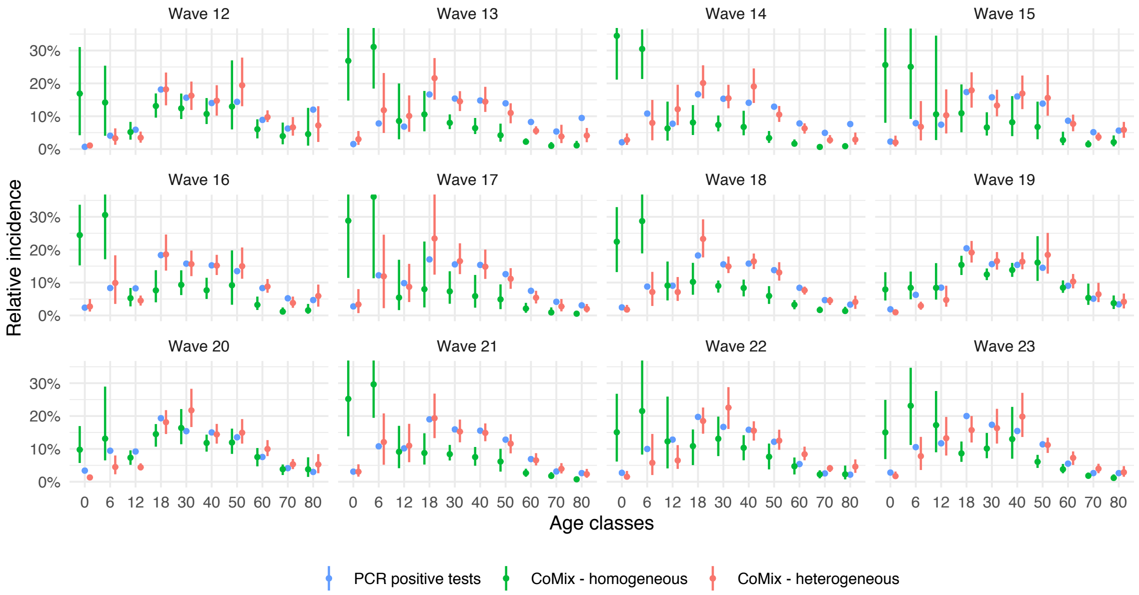


**Fig E.** Relative incidence using estimated $q$-susceptibility and assumption on infectiousness $(1, 1, 1, 1, 1, 1, 1, 1, 1, 1)$.

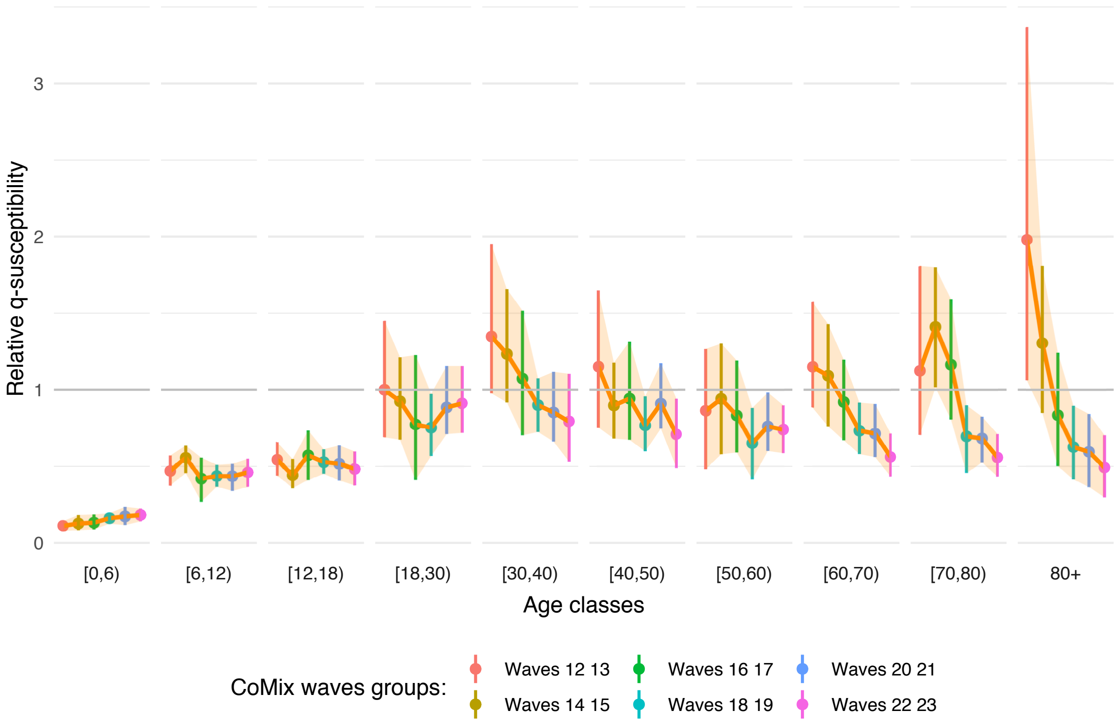


**Fig F.** Estimation of relative $q$-susceptibility with time evolution using assumption on infectiousness $(1, 1, 1, 1, 1, 1, 1, 1, 1, 1)$.

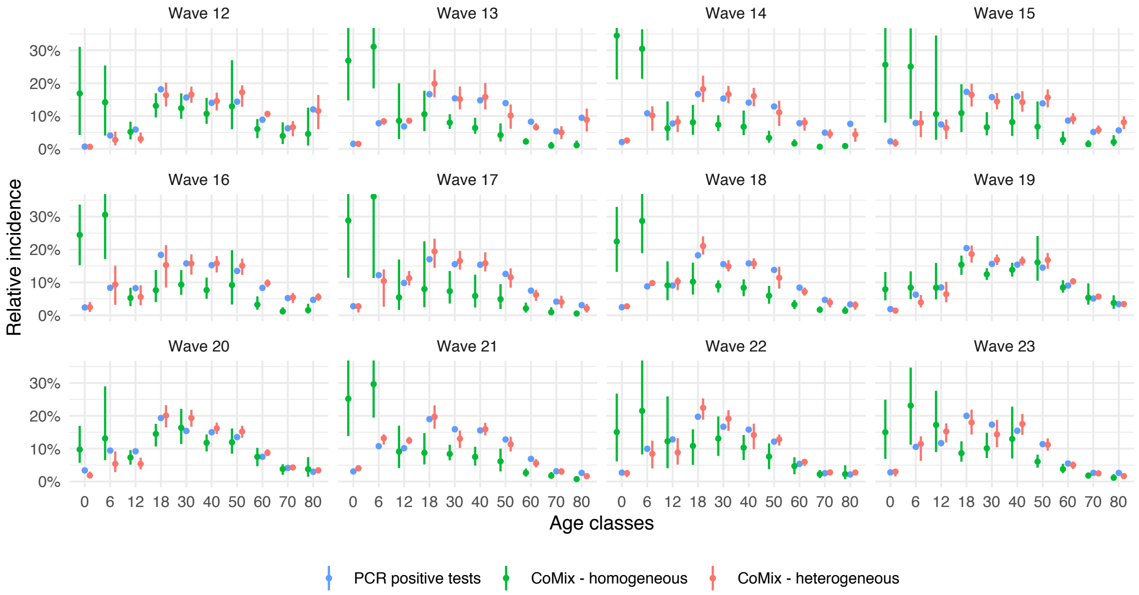


**Fig G.** Relative incidence using estimated $q$-susceptibility with time evolution and assumption on infectiousness $(1, 1, 1, 1, 1, 1, 1, 1, 1, 1)$.

| ageclass | mean | median | sd | lower | upper |
|---|---|---|---|---|---|
| [0,6) | 0.157 | 0.155 | 0.020 | 0.124 | 0.204 |
| [6,12) | 0.474 | 0.481 | 0.045 | 0.367 | 0.542 |
| [12,18) | 0.540 | 0.535 | 0.039 | 0.478 | 0.619 |
| [18,30) | 1.000 | 0.993 | 0.103 | 0.825 | 1.238 |
| [30,40) | 1.161 | 1.149 | 0.122 | 0.973 | 1.443 |
| [40,50) | 1.003 | 0.992 | 0.100 | 0.846 | 1.256 |
| [50,60) | 0.897 | 0.889 | 0.083 | 0.765 | 1.063 |
| [60,70) | 0.936 | 0.935 | 0.073 | 0.806 | 1.100 |
| [70,80) | 0.953 | 0.951 | 0.095 | 0.768 | 1.150 |
| 80+ | 0.917 | 0.914 | 0.115 | 0.695 | 1.167 |

**Table C.** Relative $q$-susceptibility using assumption on infectiousness $(1,1,1,1,1,1,1,1,1,1)$ corresponding to Figure D.

| ageclass | mean | median | sd | lower | upper |
|---|---|---|---|---|---|
| [0,6) | 0.112 | 0.112 | 0.015 | 0.081 | 0.143 |
| [6,12) | 0.469 | 0.470 | 0.053 | 0.374 | 0.571 |
| [12,18) | 0.543 | 0.543 | 0.057 | 0.438 | 0.659 |
| [18,30) | 1.000 | 0.966 | 0.201 | 0.691 | 1.449 |
| [30,40) | 1.347 | 1.314 | 0.225 | 0.978 | 1.949 |
| [40,50) | 1.150 | 1.114 | 0.240 | 0.755 | 1.649 |
| [50,60) | 0.864 | 0.862 | 0.211 | 0.481 | 1.265 |
| [60,70) | 1.149 | 1.114 | 0.178 | 0.885 | 1.573 |
| [70,80) | 1.123 | 1.113 | 0.258 | 0.706 | 1.806 |
| 80+ | 1.979 | 1.885 | 0.583 | 1.062 | 3.367 |

**(a)** Waves 12 13

| ageclass | mean | median | sd | lower | upper |
|---|---|---|---|---|---|
| [0,6) | 0.128 | 0.125 | 0.024 | 0.084 | 0.183 |
| [6,12) | 0.555 | 0.561 | 0.048 | 0.455 | 0.636 |
| [12,18) | 0.441 | 0.437 | 0.051 | 0.360 | 0.548 |
| [18,30) | 0.926 | 0.912 | 0.137 | 0.675 | 1.210 |
| [30,40) | 1.232 | 1.219 | 0.174 | 0.918 | 1.657 |
| [40,50) | 0.899 | 0.890 | 0.124 | 0.683 | 1.176 |
| [50,60) | 0.942 | 0.949 | 0.183 | 0.579 | 1.300 |
| [60,70) | 1.091 | 1.087 | 0.171 | 0.762 | 1.427 |
| [70,80) | 1.409 | 1.393 | 0.199 | 1.016 | 1.797 |
| 80+ | 1.303 | 1.286 | 0.238 | 0.850 | 1.806 |

**(b)** Waves 14 15

| ageclass | mean | median | sd | lower | upper |
|---|---|---|---|---|---|
| [0,6) | 0.133 | 0.131 | 0.025 | 0.089 | 0.186 |
| [6,12) | 0.419 | 0.424 | 0.073 | 0.270 | 0.556 |
| [12,18) | 0.572 | 0.568 | 0.084 | 0.409 | 0.735 |
| [18,30) | 0.775 | 0.761 | 0.218 | 0.410 | 1.225 |
| [30,40) | 1.072 | 1.053 | 0.217 | 0.704 | 1.516 |
| [40,50) | 0.944 | 0.926 | 0.169 | 0.675 | 1.314 |
| [50,60) | 0.833 | 0.826 | 0.153 | 0.591 | 1.189 |
| [60,70) | 0.921 | 0.917 | 0.133 | 0.671 | 1.195 |
| [70,80) | 1.162 | 1.149 | 0.202 | 0.807 | 1.592 |
| 80+ | 0.835 | 0.826 | 0.194 | 0.501 | 1.240 |

**(c)** Waves 16 17

| ageclass | mean | median | sd | lower | upper |
|---|---|---|---|---|---|
| [0,6) | 0.162 | 0.161 | 0.018 | 0.126 | 0.194 |
| [6,12) | 0.435 | 0.437 | 0.035 | 0.368 | 0.509 |
| [12,18) | 0.527 | 0.526 | 0.041 | 0.451 | 0.612 |
| [18,30) | 0.753 | 0.756 | 0.111 | 0.568 | 0.974 |
| [30,40) | 0.901 | 0.898 | 0.098 | 0.726 | 1.074 |
| [40,50) | 0.769 | 0.761 | 0.095 | 0.599 | 0.958 |
| [50,60) | 0.652 | 0.643 | 0.129 | 0.415 | 0.883 |
| [60,70) | 0.734 | 0.731 | 0.087 | 0.580 | 0.918 |
| [70,80) | 0.697 | 0.694 | 0.125 | 0.456 | 0.900 |
| 80+ | 0.626 | 0.613 | 0.129 | 0.413 | 0.897 |

**(d)** Waves 18 19

| ageclass | mean | median | sd | lower | upper |
|---|---|---|---|---|---|
| [0,6) | 0.173 | 0.172 | 0.030 | 0.117 | 0.235 |
| [6,12) | 0.434 | 0.434 | 0.049 | 0.339 | 0.515 |
| [12,18) | 0.517 | 0.517 | 0.063 | 0.406 | 0.638 |
| [18,30) | 0.886 | 0.869 | 0.120 | 0.712 | 1.154 |
| [30,40) | 0.853 | 0.839 | 0.121 | 0.663 | 1.117 |
| [40,50) | 0.911 | 0.904 | 0.112 | 0.747 | 1.172 |
| [50,60) | 0.762 | 0.754 | 0.104 | 0.601 | 0.983 |
| [60,70) | 0.715 | 0.712 | 0.091 | 0.560 | 0.909 |
| [70,80) | 0.683 | 0.683 | 0.078 | 0.525 | 0.826 |
| 80+ | 0.595 | 0.601 | 0.126 | 0.366 | 0.843 |

**(e)** Waves 20 21

| ageclass | mean | median | sd | lower | upper |
|---|---|---|---|---|---|
| [0,6) | 0.183 | 0.182 | 0.021 | 0.141 | 0.224 |
| [6,12) | 0.460 | 0.460 | 0.047 | 0.367 | 0.550 |
| [12,18) | 0.482 | 0.482 | 0.058 | 0.376 | 0.597 |
| [18,30) | 0.912 | 0.904 | 0.112 | 0.720 | 1.154 |
| [30,40) | 0.794 | 0.784 | 0.153 | 0.530 | 1.103 |
| [40,50) | 0.710 | 0.710 | 0.125 | 0.490 | 0.943 |
| [50,60) | 0.740 | 0.745 | 0.088 | 0.586 | 0.899 |
| [60,70) | 0.561 | 0.557 | 0.070 | 0.433 | 0.715 |
| [70,80) | 0.557 | 0.553 | 0.074 | 0.432 | 0.712 |
| 80+ | 0.492 | 0.486 | 0.101 | 0.297 | 0.704 |

**(f)** Waves 22 23

**Table D.** Relative $q$-susceptibility with time evolution using assumption on infectiousness $(1,1,1,1,1,1,1,1,1,1)$ corresponding to Figure F.

## Estimation of $q$-susceptibility using heterogeneous infectiousness

Method: Estimation of the $(a_i)$ relative $q$-susceptibility vector.

Assumption: heterogeneous infectiousness $(h_j) = (0.54, 0.55, 0.56, 0.59, 0.7, 0.76, 0.9, 0.99, 0.99, 0.99)$ using the proportion of asymptomatic cases in the Belgian population with asymptomatic infectiousness assumed at 0.51 as used in [2] using data from [3].

Normalization method: Mean $q$-susceptibility among children age classes [0,6), [6,12) and [12,18) is assumed constant among bootstraps and wave groups (if applicable). The mean of the first adult age class [18,30) is set to 1 for the first period.

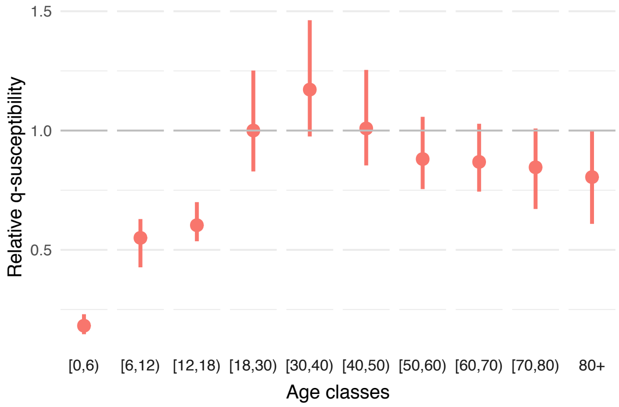


**Fig H.** Estimation of relative $q$-susceptibility using assumption on infectiousness $(0.54, 0.55, 0.56, 0.59, 0.7, 0.76, 0.9, 0.99, 0.99, 0.99)$.

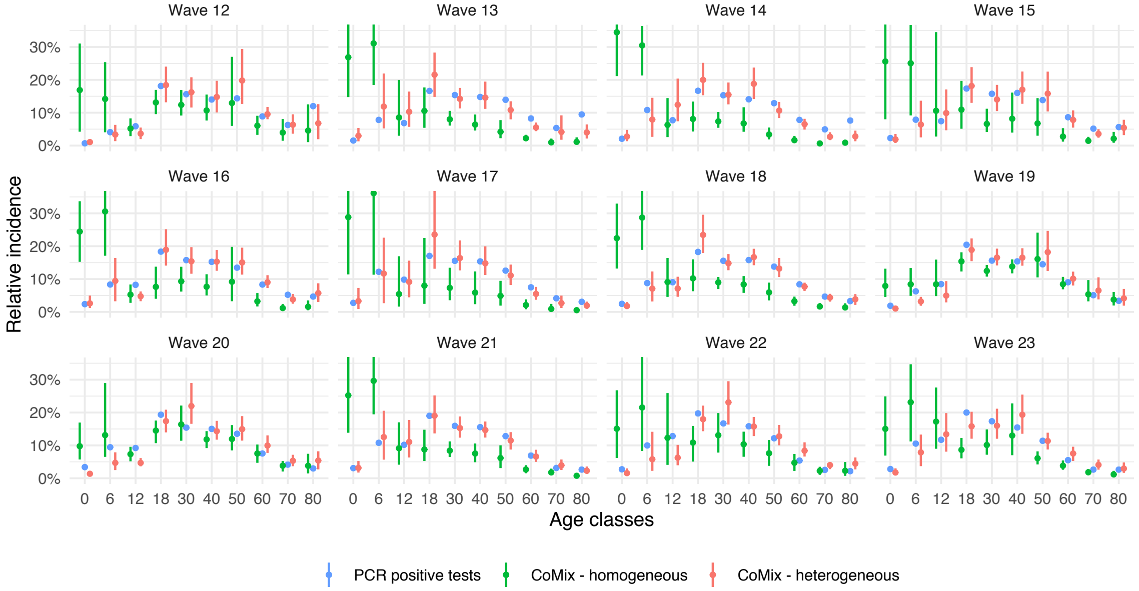


**Fig I.** Relative incidence using estimated $q$-susceptibility and assumption on infectiousness $(0.54, 0.55, 0.56, 0.59, 0.7, 0.76, 0.9, 0.99, 0.99, 0.99)$.

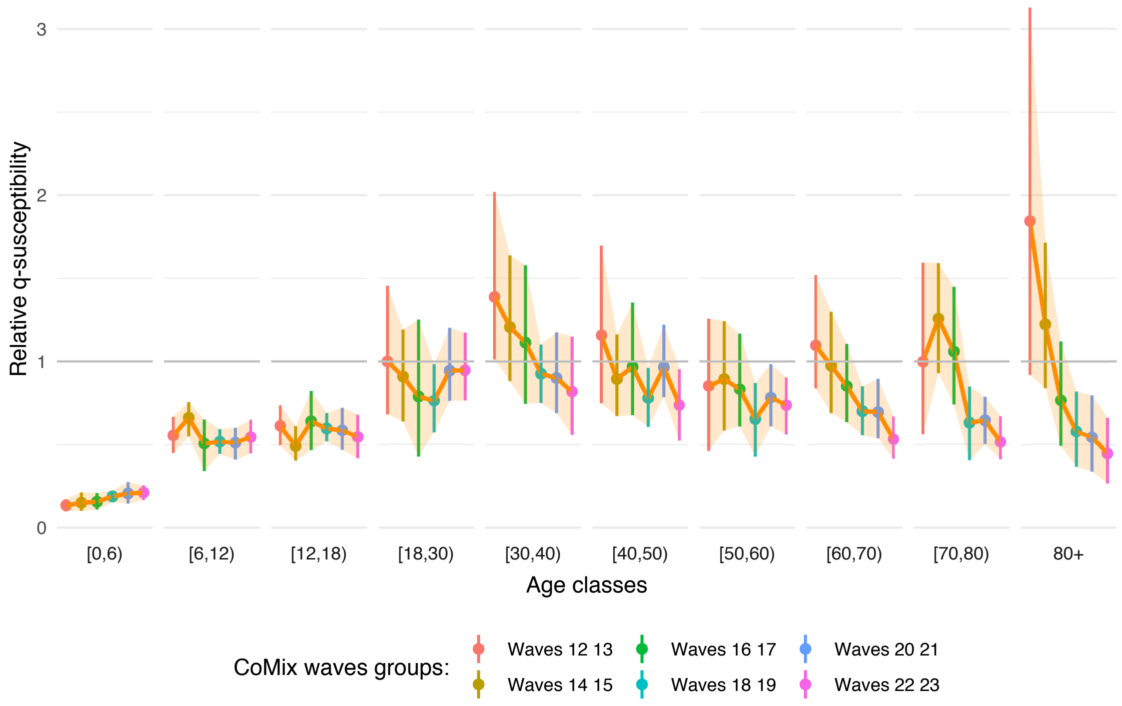


**Fig J.** Estimation of relative $q$-susceptibility with time evolution using assumption on infectiousness $(0.54, 0.55, 0.56, 0.59, 0.7, 0.76, 0.9, 0.99, 0.99, 0.99)$.

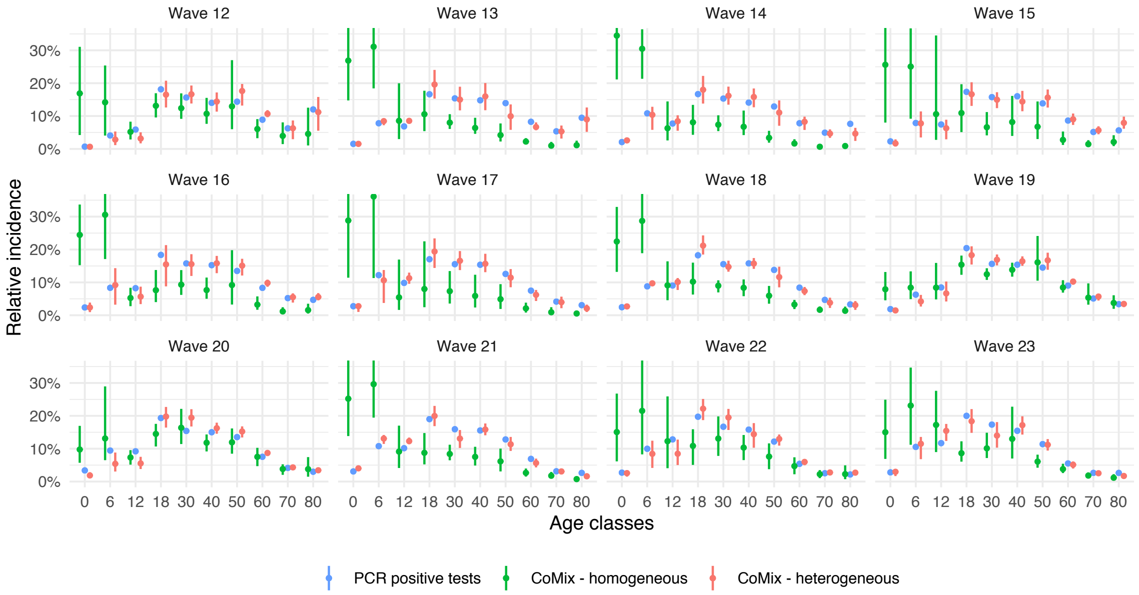


**Fig K.** Relative incidence using estimated $q$-susceptibility with time evolution and assumption on infectiousness $(0.54, 0.55, 0.56, 0.59, 0.7, 0.76, 0.9, 0.99, 0.99, 0.99)$.

| ageclass | mean | median | sd | lower | upper |
|---|---|---|---|---|---|
| [0,6) | 0.182 | 0.180 | 0.020 | 0.146 | 0.230 |
| [6,12) | 0.550 | 0.559 | 0.051 | 0.427 | 0.629 |
| [12,18) | 0.603 | 0.599 | 0.044 | 0.536 | 0.700 |
| [18,30) | 1.000 | 0.993 | 0.102 | 0.829 | 1.252 |
| [30,40) | 1.172 | 1.162 | 0.120 | 0.975 | 1.462 |
| [40,50) | 1.009 | 0.997 | 0.100 | 0.854 | 1.254 |
| [50,60) | 0.880 | 0.875 | 0.082 | 0.755 | 1.057 |
| [60,70) | 0.869 | 0.864 | 0.070 | 0.744 | 1.028 |
| [70,80) | 0.846 | 0.849 | 0.090 | 0.671 | 1.009 |
| 80+ | 0.805 | 0.798 | 0.101 | 0.609 | 1.000 |

**Table E.** Relative $q$-susceptibility using assumption on infectiousness $(0.54, 0.55, 0.56, 0.59, 0.7, 0.76, 0.9, 0.99, 0.99, 0.99)$ corresponding to Figure H.

| ageclass | mean | median | sd | lower | upper |
|---|---|---|---|---|---|
| [0,6) | 0.134 | 0.134 | 0.018 | 0.103 | 0.172 |
| [6,12) | 0.555 | 0.556 | 0.059 | 0.449 | 0.666 |
| [12,18) | 0.613 | 0.616 | 0.064 | 0.495 | 0.735 |
| [18,30) | 1.000 | 0.971 | 0.208 | 0.680 | 1.454 |
| [30,40) | 1.387 | 1.346 | 0.238 | 1.012 | 2.019 |
| [40,50) | 1.157 | 1.125 | 0.249 | 0.746 | 1.695 |
| [50,60) | 0.853 | 0.853 | 0.216 | 0.461 | 1.255 |
| [60,70) | 1.096 | 1.066 | 0.169 | 0.839 | 1.519 |
| [70,80) | 0.999 | 0.976 | 0.249 | 0.563 | 1.593 |
| 80+ | 1.844 | 1.763 | 0.567 | 0.920 | 3.127 |

**(a)** Waves 12 13

| ageclass | mean | median | sd | lower | upper |
|---|---|---|---|---|---|
| [0,6) | 0.151 | 0.148 | 0.027 | 0.103 | 0.212 |
| [6,12) | 0.660 | 0.663 | 0.056 | 0.548 | 0.753 |
| [12,18) | 0.491 | 0.484 | 0.058 | 0.402 | 0.611 |
| [18,30) | 0.911 | 0.912 | 0.136 | 0.639 | 1.191 |
| [30,40) | 1.205 | 1.189 | 0.178 | 0.883 | 1.637 |
| [40,50) | 0.896 | 0.888 | 0.123 | 0.670 | 1.161 |
| [50,60) | 0.895 | 0.893 | 0.177 | 0.585 | 1.241 |
| [60,70) | 0.975 | 0.980 | 0.155 | 0.687 | 1.299 |
| [70,80) | 1.258 | 1.257 | 0.181 | 0.931 | 1.590 |
| 80+ | 1.222 | 1.198 | 0.231 | 0.841 | 1.715 |

**(b)** Waves 14 15

| ageclass | mean | median | sd | lower | upper |
|---|---|---|---|---|---|
| [0,6) | 0.157 | 0.157 | 0.026 | 0.110 | 0.209 |
| [6,12) | 0.506 | 0.513 | 0.081 | 0.341 | 0.650 |
| [12,18) | 0.639 | 0.630 | 0.093 | 0.466 | 0.822 |
| [18,30) | 0.789 | 0.785 | 0.221 | 0.428 | 1.250 |
| [30,40) | 1.113 | 1.089 | 0.222 | 0.742 | 1.577 |
| [40,50) | 0.968 | 0.957 | 0.170 | 0.676 | 1.353 |
| [50,60) | 0.834 | 0.825 | 0.148 | 0.609 | 1.166 |
| [60,70) | 0.853 | 0.854 | 0.122 | 0.634 | 1.105 |
| [70,80) | 1.060 | 1.041 | 0.187 | 0.739 | 1.446 |
| 80+ | 0.765 | 0.746 | 0.159 | 0.491 | 1.119 |

**(c)** Waves 16 17

| ageclass | mean | median | sd | lower | upper |
|---|---|---|---|---|---|
| [0,6) | 0.189 | 0.188 | 0.018 | 0.153 | 0.224 |
| [6,12) | 0.516 | 0.520 | 0.039 | 0.444 | 0.593 |
| [12,18) | 0.597 | 0.593 | 0.044 | 0.517 | 0.688 |
| [18,30) | 0.763 | 0.761 | 0.113 | 0.574 | 0.984 |
| [30,40) | 0.927 | 0.925 | 0.097 | 0.748 | 1.101 |
| [40,50) | 0.779 | 0.775 | 0.093 | 0.606 | 0.961 |
| [50,60) | 0.654 | 0.645 | 0.123 | 0.428 | 0.872 |
| [60,70) | 0.699 | 0.701 | 0.078 | 0.555 | 0.852 |
| [70,80) | 0.632 | 0.640 | 0.122 | 0.405 | 0.851 |
| 80+ | 0.578 | 0.577 | 0.122 | 0.365 | 0.819 |

**(d)** Waves 18 19

| ageclass | mean | median | sd | lower | upper |
|---|---|---|---|---|---|
| [0,6) | 0.206 | 0.204 | 0.033 | 0.146 | 0.274 |
| [6,12) | 0.511 | 0.513 | 0.055 | 0.407 | 0.601 |
| [12,18) | 0.585 | 0.588 | 0.069 | 0.467 | 0.719 |
| [18,30) | 0.946 | 0.936 | 0.119 | 0.763 | 1.201 |
| [30,40) | 0.902 | 0.900 | 0.129 | 0.688 | 1.174 |
| [40,50) | 0.965 | 0.953 | 0.115 | 0.785 | 1.219 |
| [50,60) | 0.782 | 0.776 | 0.102 | 0.610 | 0.984 |
| [60,70) | 0.694 | 0.691 | 0.091 | 0.537 | 0.896 |
| [70,80) | 0.645 | 0.651 | 0.077 | 0.502 | 0.787 |
| 80+ | 0.543 | 0.530 | 0.122 | 0.338 | 0.796 |

**(e)** Waves 20 21

| ageclass | mean | median | sd | lower | upper |
|---|---|---|---|---|---|
| [0,6) | 0.211 | 0.210 | 0.023 | 0.167 | 0.255 |
| [6,12) | 0.545 | 0.545 | 0.054 | 0.447 | 0.649 |
| [12,18) | 0.546 | 0.546 | 0.066 | 0.417 | 0.678 |
| [18,30) | 0.948 | 0.929 | 0.111 | 0.766 | 1.172 |
| [30,40) | 0.819 | 0.810 | 0.153 | 0.559 | 1.149 |
| [40,50) | 0.737 | 0.735 | 0.122 | 0.524 | 0.954 |
| [50,60) | 0.737 | 0.736 | 0.088 | 0.562 | 0.905 |
| [60,70) | 0.532 | 0.530 | 0.067 | 0.414 | 0.668 |
| [70,80) | 0.516 | 0.507 | 0.071 | 0.410 | 0.669 |
| 80+ | 0.446 | 0.439 | 0.099 | 0.266 | 0.660 |

**(f)** Waves 22 23

**Table F.** Relative $q$-susceptibility with time evolution using assumption on infectiousness $(0.54, 0.55, 0.56, 0.59, 0.7, 0.76, 0.9, 0.99, 0.99, 0.99)$ corresponding to Figure J.

## Estimation of $q$-infectiousness using homogeneous susceptibility

Method: Estimation of the $(h_j)$ relative $q$-infectiousness vector.

Assumption: homogeneous susceptibility $(a_i) = (1, 1, 1, 1, 1, 1, 1, 1, 1, 1)$.

Normalization method: No normalization accross bootstraps and wave groups is applied here since the $q$-infectiousness among children age classes is estimated at $(0, 0, 0)$ for several bootstraps and this prevents using the same normaliztion method than in other subsections. The mean of the first adult age class [18,30) is set to 1 for the first period.

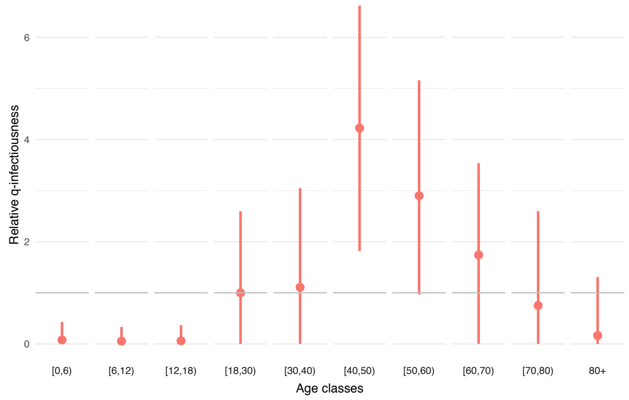


**Fig L.** Estimation of relative $q$-infectiousness using assumption on susceptibility $(1, 1, 1, 1, 1, 1, 1, 1, 1, 1)$.

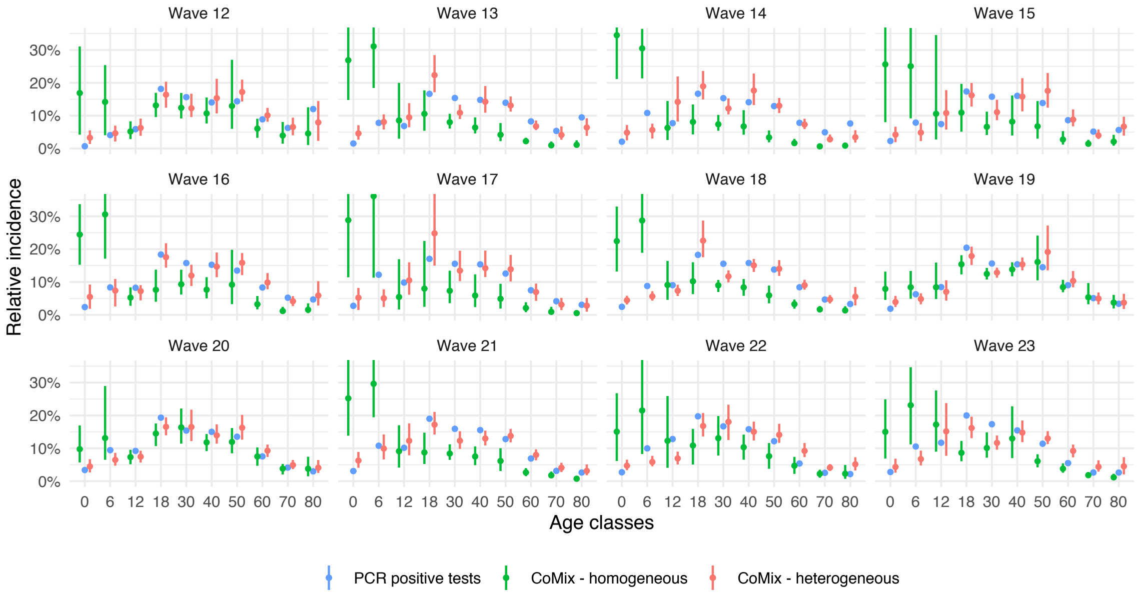


**Fig M.** Relative incidence using estimated $q$-infectiousness and assumption on susceptibility $(1, 1, 1, 1, 1, 1, 1, 1, 1, 1)$.

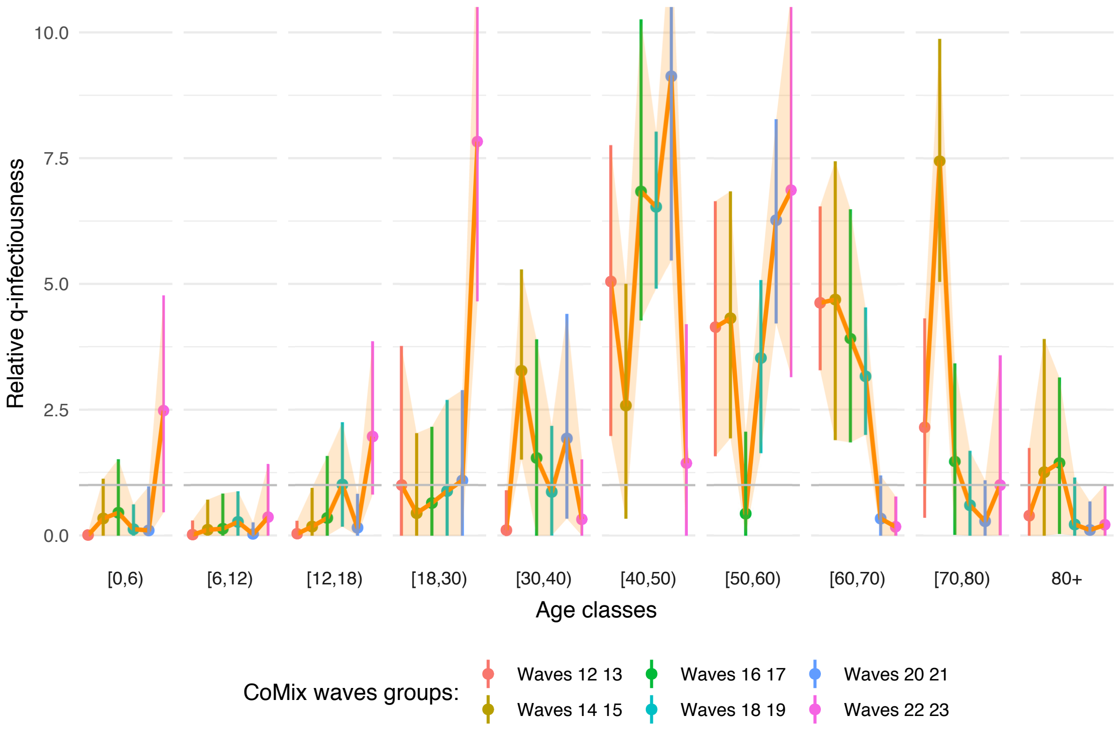


**Fig N.** Estimation of relative $q$-infectiousness with time evolution using assumption on susceptibility $(1, 1, 1, 1, 1, 1, 1, 1, 1, 1)$.

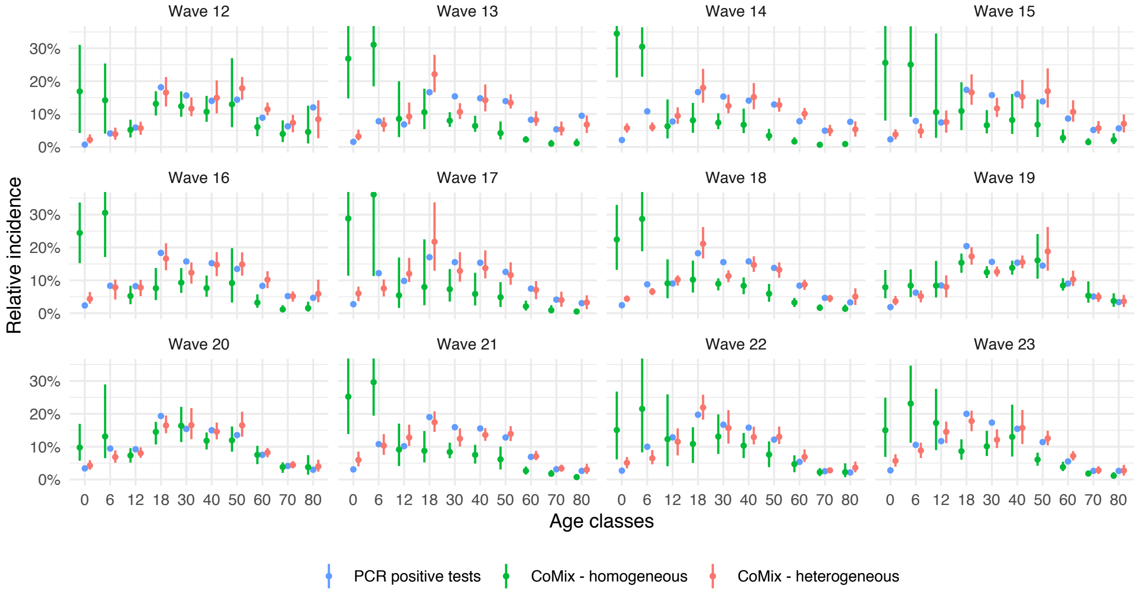


**Fig O.** Relative incidence using estimated $q$-infectiousness with time evolution and assumption on susceptibility $(1, 1, 1, 1, 1, 1, 1, 1, 1, 1)$.

| ageclass | mean | median | sd | lower | upper |
|---|---|---|---|---|---|
| [0,6) | 0.078 | 0.000 | 0.129 | 0.000 | 0.430 |
| [6,12) | 0.054 | 0.000 | 0.111 | 0.000 | 0.331 |
| [12,18) | 0.060 | 0.000 | 0.106 | 0.000 | 0.368 |
| [18,30) | 1.000 | 0.872 | 0.733 | 0.000 | 2.594 |
| [30,40) | 1.108 | 0.991 | 0.910 | 0.000 | 3.049 |
| [40,50) | 4.224 | 4.220 | 1.176 | 1.818 | 6.619 |
| [50,60) | 2.899 | 2.896 | 1.144 | 0.968 | 5.159 |
| [60,70) | 1.741 | 1.694 | 0.882 | 0.000 | 3.536 |
| [70,80) | 0.752 | 0.513 | 0.810 | 0.000 | 2.598 |
| 80+ | 0.164 | 0.000 | 0.372 | 0.000 | 1.307 |

**Table G.** Relative $q$-infectiousness using assumption on susceptibility $(1,1,1,1,1,1,1,1,1,1)$ corresponding to Figure L.

| ageclass | mean | median | sd | lower | upper |
|---|---|---|---|---|---|
| [0,6) | 0.009 | 0.000 | 0.034 | 0.000 | 0.071 |
| [6,12) | 0.018 | 0.000 | 0.086 | 0.000 | 0.300 |
| [12,18) | 0.036 | 0.000 | 0.103 | 0.000 | 0.295 |
| [18,30) | 1.000 | 0.639 | 1.136 | 0.000 | 3.763 |
| [30,40) | 0.108 | 0.012 | 0.232 | 0.000 | 0.897 |
| [40,50) | 5.047 | 5.173 | 1.413 | 1.977 | 7.763 |
| [50,60) | 4.143 | 4.061 | 1.254 | 1.574 | 6.643 |
| [60,70) | 4.625 | 4.608 | 0.839 | 3.281 | 6.540 |
| [70,80) | 2.149 | 2.073 | 1.013 | 0.353 | 4.318 |
| 80+ | 0.394 | 0.154 | 0.556 | 0.000 | 1.739 |

**(a)** Waves 12 13

| ageclass | mean | median | sd | lower | upper |
|---|---|---|---|---|---|
| [0,6) | 0.340 | 0.267 | 0.330 | 0.000 | 1.124 |
| [6,12) | 0.117 | 0.023 | 0.214 | 0.000 | 0.711 |
| [12,18) | 0.180 | 0.029 | 0.271 | 0.000 | 0.943 |
| [18,30) | 0.445 | 0.147 | 0.632 | 0.000 | 2.034 |
| [30,40) | 3.270 | 3.137 | 1.012 | 1.508 | 5.281 |
| [40,50) | 2.581 | 2.458 | 1.152 | 0.335 | 5.005 |
| [50,60) | 4.321 | 4.263 | 1.242 | 1.930 | 6.838 |
| [60,70) | 4.689 | 4.919 | 1.575 | 1.894 | 7.435 |
| [70,80) | 7.442 | 7.427 | 1.299 | 5.041 | 9.871 |
| 80+ | 1.252 | 1.047 | 1.024 | 0.000 | 3.903 |

**(b)** Waves 14 15

| ageclass | mean | median | sd | lower | upper |
|---|---|---|---|---|---|
| [0,6) | 0.453 | 0.359 | 0.474 | 0.000 | 1.516 |
| [6,12) | 0.137 | 0.004 | 0.236 | 0.000 | 0.831 |
| [12,18) | 0.350 | 0.172 | 0.468 | 0.000 | 1.580 |
| [18,30) | 0.644 | 0.566 | 0.581 | 0.000 | 2.159 |
| [30,40) | 1.541 | 1.399 | 1.168 | 0.000 | 3.898 |
| [40,50) | 6.836 | 6.716 | 1.669 | 4.277 | 10.259 |
| [50,60) | 0.436 | 0.114 | 0.613 | 0.000 | 2.063 |
| [60,70) | 3.914 | 3.893 | 1.357 | 1.850 | 6.483 |
| [70,80) | 1.468 | 1.447 | 0.988 | 0.016 | 3.418 |
| 80+ | 1.439 | 1.362 | 0.810 | 0.034 | 3.138 |

**(c)** Waves 16 17

| ageclass | mean | median | sd | lower | upper |
|---|---|---|---|---|---|
| [0,6) | 0.128 | 0.042 | 0.180 | 0.000 | 0.618 |
| [6,12) | 0.272 | 0.227 | 0.238 | 0.000 | 0.876 |
| [12,18) | 1.008 | 0.938 | 0.523 | 0.178 | 2.250 |
| [18,30) | 0.880 | 0.649 | 0.870 | 0.000 | 2.692 |
| [30,40) | 0.864 | 0.774 | 0.601 | 0.003 | 2.178 |
| [40,50) | 6.531 | 6.746 | 0.835 | 4.912 | 8.031 |
| [50,60) | 3.524 | 3.749 | 0.945 | 1.635 | 5.077 |
| [60,70) | 3.161 | 3.088 | 0.694 | 1.998 | 4.534 |
| [70,80) | 0.600 | 0.447 | 0.542 | 0.000 | 1.683 |
| 80+ | 0.221 | 0.084 | 0.315 | 0.000 | 1.147 |

**(d)** Waves 18 19

| ageclass | mean | median | sd | lower | upper |
|---|---|---|---|---|---|
| [0,6) | 0.105 | 0.001 | 0.268 | 0.000 | 0.971 |
| [6,12) | 0.031 | 0.000 | 0.112 | 0.000 | 0.263 |
| [12,18) | 0.158 | 0.026 | 0.254 | 0.000 | 0.826 |
| [18,30) | 1.088 | 0.833 | 0.897 | 0.000 | 2.888 |
| [30,40) | 1.928 | 1.887 | 0.924 | 0.336 | 4.407 |
| [40,50) | 9.128 | 9.524 | 1.643 | 5.465 | 12.172 |
| [50,60) | 6.266 | 6.296 | 1.093 | 4.218 | 8.278 |
| [60,70) | 0.338 | 0.178 | 0.386 | 0.000 | 1.188 |
| [70,80) | 0.285 | 0.156 | 0.307 | 0.000 | 1.093 |
| 80+ | 0.113 | 0.029 | 0.263 | 0.000 | 0.675 |

**(e)** Waves 20 21

| ageclass | mean | median | sd | lower | upper |
|---|---|---|---|---|---|
| [0,6) | 2.481 | 2.357 | 1.230 | 0.461 | 4.773 |
| [6,12) | 0.365 | 0.219 | 0.450 | 0.000 | 1.416 |
| [12,18) | 1.966 | 1.842 | 0.794 | 0.811 | 3.857 |
| [18,30) | 7.831 | 7.097 | 2.411 | 4.653 | 12.346 |
| [30,40) | 0.322 | 0.108 | 0.561 | 0.000 | 1.513 |
| [40,50) | 1.436 | 1.264 | 1.076 | 0.000 | 4.204 |
| [50,60) | 6.863 | 7.341 | 2.154 | 3.143 | 10.833 |
| [60,70) | 0.174 | 0.079 | 0.305 | 0.000 | 0.773 |
| [70,80) | 0.999 | 0.672 | 0.992 | 0.007 | 3.578 |
| 80+ | 0.214 | 0.104 | 0.274 | 0.000 | 1.010 |

**(f)** Waves 22 23

**Table H.** Relative $q$-infectiousness with time evolution using assumption on susceptibility $(1,1,1,1,1,1,1,1,1,1)$ corresponding to Figure N.

## Estimation of $q$-infectiousness using heterogeneous susceptibility

Method: Estimation of the $(h_j)$ relative $q$-infectiousness vector.

Assumption: heterogeneous susceptibility $(a_i) = (0.4, 0.39, 0.38, 0.79, 0.86, 0.8, 0.82, 0.88, 0.74, 0.74)$ taken from [4].

Normalization method: Mean $q$-infectiousness among children age classes [0,6), [6,12) and [12,18) is assumed constant among bootstraps and wave groups (if applicable). The mean of the first adult age class [18,30) is set to 1 for the first period.

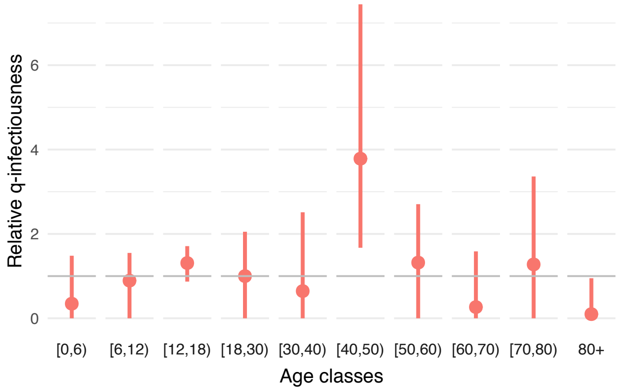


**Fig P.** Estimation of relative $q$-infectiousness using assumption on susceptibility $(0.4, 0.39, 0.38, 0.79, 0.86, 0.8, 0.82, 0.88, 0.74, 0.74)$.

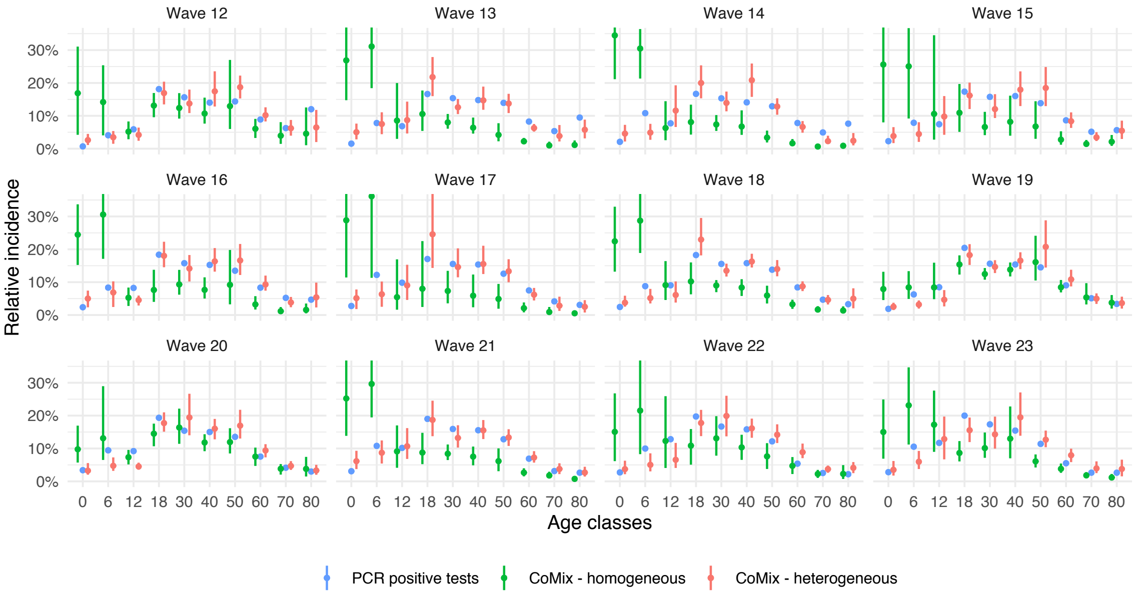


**Fig Q.** Relative incidence using estimated $q$-infectiousness and assumption on susceptibility $(0.4, 0.39, 0.38, 0.79, 0.86, 0.8, 0.82, 0.88, 0.74, 0.74)$.

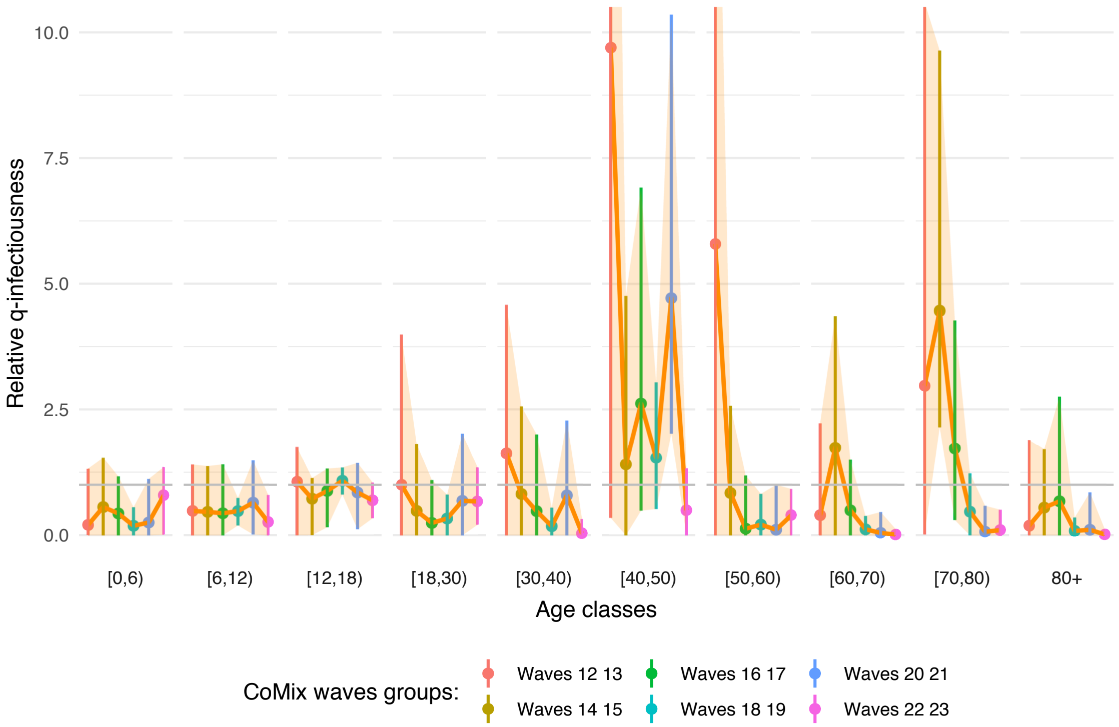


**Fig R.** Estimation of relative $q$-infectiousness with time evolution using assumption on susceptibility $(0.4, 0.39, 0.38, 0.79, 0.86, 0.8, 0.82, 0.88, 0.74, 0.74)$.

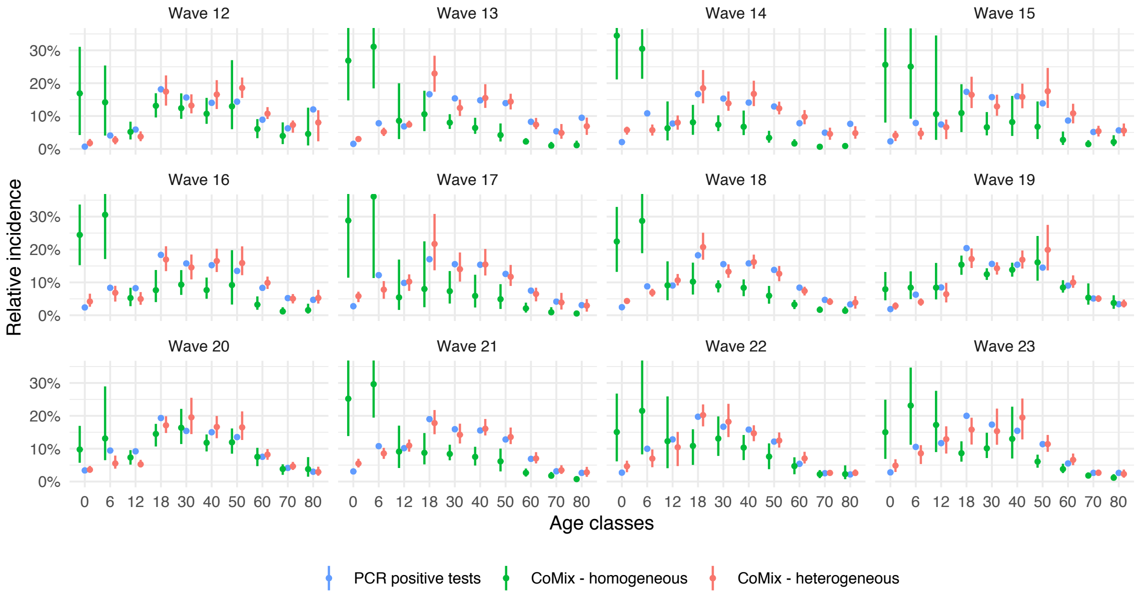


**Fig S.** Relative incidence using estimated $q$-infectiousness with time evolution and assumption on susceptibility $(0.4, 0.39, 0.38, 0.79, 0.86, 0.8, 0.82, 0.88, 0.74, 0.74)$.

| ageclass | mean | median | sd | lower | upper |
|---|---|---|---|---|---|
| [0,6) | 0.346 | 0.034 | 0.461 | 0.000 | 1.483 |
| [6,12) | 0.892 | 0.957 | 0.404 | 0.000 | 1.549 |
| [12,18) | 1.310 | 1.326 | 0.231 | 0.870 | 1.710 |
| [18,30) | 1.000 | 0.967 | 0.544 | 0.000 | 2.051 |
| [30,40) | 0.645 | 0.375 | 0.811 | 0.000 | 2.512 |
| [40,50) | 3.783 | 3.541 | 1.586 | 1.672 | 7.443 |
| [50,60) | 1.320 | 1.257 | 0.741 | 0.000 | 2.705 |
| [60,70) | 0.266 | 0.000 | 0.445 | 0.000 | 1.586 |
| [70,80) | 1.277 | 1.155 | 0.903 | 0.000 | 3.362 |
| 80+ | 0.099 | 0.000 | 0.279 | 0.000 | 0.950 |

**Table I.** Relative $q$-infectiousness using assumption on susceptibility $(0.4, 0.39, 0.38, 0.79, 0.86, 0.8, 0.82, 0.88, 0.74, 0.74)$ corresponding to Figure P.

| ageclass | mean | median | sd | lower | upper |
|---|---|---|---|---|---|
| [0,6) | 0.204 | 0.013 | 0.363 | 0.000 | 1.319 |
| [6,12) | 0.483 | 0.454 | 0.383 | 0.000 | 1.402 |
| [12,18) | 1.061 | 1.070 | 0.443 | 0.001 | 1.748 |
| [18,30) | 1.000 | 0.625 | 1.259 | 0.000 | 3.990 |
| [30,40) | 1.625 | 0.052 | 12.906 | 0.000 | 4.577 |
| [40,50) | 9.694 | 3.895 | 49.732 | 0.345 | 25.974 |
| [50,60) | 5.792 | 2.434 | 31.815 | 0.000 | 17.774 |
| [60,70) | 0.398 | 0.063 | 0.802 | 0.000 | 2.223 |
| [70,80) | 2.973 | 2.087 | 4.053 | 0.018 | 10.597 |
| 80+ | 0.189 | 0.000 | 0.505 | 0.000 | 1.891 |

**(a)** Waves 12 13

| ageclass | mean | median | sd | lower | upper |
|---|---|---|---|---|---|
| [0,6) | 0.560 | 0.547 | 0.453 | 0.000 | 1.535 |
| [6,12) | 0.464 | 0.404 | 0.436 | 0.000 | 1.370 |
| [12,18) | 0.724 | 0.760 | 0.278 | 0.005 | 1.134 |
| [18,30) | 0.484 | 0.287 | 0.554 | 0.000 | 1.814 |
| [30,40) | 0.818 | 0.700 | 0.695 | 0.000 | 2.559 |
| [40,50) | 1.404 | 1.172 | 1.127 | 0.000 | 4.761 |
| [50,60) | 0.838 | 0.739 | 0.711 | 0.000 | 2.570 |
| [60,70) | 1.737 | 1.647 | 1.033 | 0.000 | 4.353 |
| [70,80) | 4.463 | 4.026 | 1.995 | 2.142 | 9.635 |
| 80+ | 0.549 | 0.501 | 0.550 | 0.000 | 1.707 |

**(b)** Waves 14 15

| ageclass | mean | median | sd | lower | upper |
|---|---|---|---|---|---|
| [0,6) | 0.433 | 0.425 | 0.412 | 0.000 | 1.167 |
| [6,12) | 0.436 | 0.388 | 0.398 | 0.000 | 1.405 |
| [12,18) | 0.879 | 0.897 | 0.267 | 0.161 | 1.322 |
| [18,30) | 0.246 | 0.153 | 0.311 | 0.000 | 1.093 |
| [30,40) | 0.479 | 0.212 | 0.661 | 0.000 | 2.002 |
| [40,50) | 2.615 | 2.145 | 1.843 | 0.488 | 6.913 |
| [50,60) | 0.133 | 0.000 | 0.341 | 0.000 | 1.184 |
| [60,70) | 0.495 | 0.400 | 0.432 | 0.000 | 1.497 |
| [70,80) | 1.725 | 1.432 | 1.105 | 0.303 | 4.271 |
| 80+ | 0.674 | 0.501 | 0.764 | 0.000 | 2.751 |

**(c)** Waves 16 17

| ageclass | mean | median | sd | lower | upper |
|---|---|---|---|---|---|
| [0,6) | 0.189 | 0.146 | 0.177 | 0.000 | 0.556 |
| [6,12) | 0.480 | 0.484 | 0.147 | 0.196 | 0.740 |
| [12,18) | 1.079 | 1.090 | 0.142 | 0.806 | 1.342 |
| [18,30) | 0.333 | 0.320 | 0.230 | 0.000 | 0.806 |
| [30,40) | 0.181 | 0.139 | 0.178 | 0.000 | 0.548 |
| [40,50) | 1.536 | 1.418 | 0.703 | 0.520 | 3.044 |
| [50,60) | 0.212 | 0.143 | 0.235 | 0.000 | 0.820 |
| [60,70) | 0.120 | 0.083 | 0.127 | 0.000 | 0.384 |
| [70,80) | 0.469 | 0.432 | 0.328 | 0.001 | 1.227 |
| 80+ | 0.089 | 0.021 | 0.115 | 0.000 | 0.354 |

**(d)** Waves 18 19

| ageclass | mean | median | sd | lower | upper |
|---|---|---|---|---|---|
| [0,6) | 0.255 | 0.038 | 0.367 | 0.000 | 1.115 |
| [6,12) | 0.646 | 0.662 | 0.376 | 0.018 | 1.484 |
| [12,18) | 0.847 | 0.867 | 0.335 | 0.121 | 1.433 |
| [18,30) | 0.682 | 0.607 | 0.546 | 0.000 | 2.017 |
| [30,40) | 0.790 | 0.709 | 0.631 | 0.000 | 2.279 |
| [40,50) | 4.716 | 4.233 | 2.591 | 2.017 | 10.348 |
| [50,60) | 0.112 | 0.002 | 0.262 | 0.000 | 0.999 |
| [60,70) | 0.049 | 0.003 | 0.149 | 0.000 | 0.461 |
| [70,80) | 0.072 | 0.004 | 0.196 | 0.000 | 0.585 |
| 80+ | 0.111 | 0.004 | 0.248 | 0.000 | 0.851 |

**(e)** Waves 20 21

| ageclass | mean | median | sd | lower | upper |
|---|---|---|---|---|---|
| [0,6) | 0.794 | 0.823 | 0.321 | 0.013 | 1.352 |
| [6,12) | 0.263 | 0.208 | 0.247 | 0.000 | 0.799 |
| [12,18) | 0.692 | 0.689 | 0.189 | 0.339 | 1.044 |
| [18,30) | 0.671 | 0.644 | 0.274 | 0.210 | 1.346 |
| [30,40) | 0.039 | 0.000 | 0.109 | 0.000 | 0.323 |
| [40,50) | 0.496 | 0.436 | 0.404 | 0.000 | 1.327 |
| [50,60) | 0.399 | 0.391 | 0.270 | 0.000 | 0.918 |
| [60,70) | 0.014 | 0.000 | 0.042 | 0.000 | 0.123 |
| [70,80) | 0.104 | 0.053 | 0.144 | 0.000 | 0.507 |
| 80+ | 0.016 | 0.004 | 0.033 | 0.000 | 0.105 |

**(f)** Waves 22 23

**Table J.** Relative $q$-infectiousness with time evolution using assumption on susceptibility $(0.4, 0.39, 0.38, 0.79, 0.86, 0.8, 0.82, 0.88, 0.74, 0.74)$ corresponding to Figure R.

# References

1. History of Belgian testing policy and procedures; 2021. https://covid-19.sciensano.be/fr/procedures/historique-des-changements.

2. Abrams S, Wambua J, Santermans E, Willem L, Kuylen E, Coletti P, et al. Modelling the early phase of the Belgian COVID-19 epidemic using a stochastic compartmental model and studying its implied future trajectories. Epidemics. 2021;35:100449. doi:https://doi.org/10.1016/j.epidem.2021.100449.

3. Wu JT, Leung K, Bushman M, Kishore N, Niehus R, de Salazar PM, et al. Estimating clinical severity of COVID-19 from the transmission dynamics in Wuhan, China. Nature Medicine. 2020;26(4):506–510. doi:10.1038/s41591-020-0822-7.

4. Davies NG, Klepac P, Liu Y, Prem K, Jit M, Pearson CAB, et al. Age-dependent effects in the transmission and control of COVID-19 epidemics. Nature Medicine. 2020;26(8):1205–1211. doi:10.1038/s41591-020-0962-9.
